# Impact of antibiotic treatment for chronic endometritis on pregnancy outcomes in women with reproductive failures (RIF and RPL): A systematic review and meta-analysis

**DOI:** 10.3389/fmed.2022.980511

**Published:** 2022-11-03

**Authors:** Jingjing Liu, Zheng Ai Liu, Yichun Liu, Lei Cheng, Lei Yan

**Affiliations:** ^1^Center for Reproductive Medicine, Cheeloo College of Medicine, Shandong University, Jinan, Shandong, China; ^2^Maternal and Child Health Hospital of Zoucheng, Zoucheng, Shandong, China; ^3^Qilu Hospital (Qingdao), Cheeloo College of Medicine, Shandong University, Qingdao, Shandong, China

**Keywords:** chronic endometritis, infertility, antibiotic treatment, live birth rate, miscarriage rate

## Abstract

**Objective:**

The aim of this study was to investigate the effect of antibiotic treatment for chronic endometritis (CE) on reproductive outcomes.

**Design:**

Systematic review and meta-analysis.

**Patients:**

Women with reproductive failures, including recurrent implantation failure (RIF), and recurrent pregnancy loss (RPL).

**Interventions:**

Literature searches were performed using three electronic databases (PubMed, Embase, and Web of Science) until 1 December 2021 (without date restriction). The following comparators were included: women with CE receiving antibiotics vs. untreated controls; women with cured CE vs. women with normal endometrial histology (negative for CE); and women with cured CE vs. women with persistent CE (PCE). The summary measures were indicated as odds ratio (OR) with a 95% confidence interval (CI).

**Main outcome measures:**

These include on-going pregnancy rate/live birth rate (OPR/LBR), clinical pregnancy rate (CPR), and miscarriage rate/pregnancy loss rate (MR/PLR).

**Results:**

A total of 2,154 women (from twelve studies) were enrolled. Compared with the control group, women with CE receiving antibiotics did not show a statistically significant difference in OPR/LBR (*P* = 0.09) and CPR (*P* = 0.36), although there was a lower MR (*P* = 0.03). Women with cured CE have higher OPR/LBR (OR 1.57) and CPR (OR 1.56) in comparison with those with non-CE. There was a statistically significantly higher OPR/LBR (OR 6.82, *P* < 0.00001) and CPR (OR 9.75, *P* < 0.00001) in women with cured CE vs. those with persistent CE.

**Conclusion:**

While antibiotic treatment is a sensible option to cure CE, more well-designed prospective studies are needed to evaluate the reproductive impact of antibiotic treatment. Cured CE provides high-quality maternal conditions for subsequent embryo transfer and successful pregnancy.

## Introduction

Chronic endometritis (CE) is an inflammatory disease characterized by the persistent presence of plasma cells in the endometrial stroma (1). CE often shows asymptomatic or subtle clinical disturbances, which consist of abnormal uterine bleeding (AUB), pelvic pain, and leukorrhea. Nevertheless, recent emerging studies demonstrate that CE may be associated with intrauterine pathological features such as polyps or fibroids and reproductive failures including recurrent pregnancy loss (RPL) and recurrent implantation failure (RIF) (2–8).

Chronic endometritis is a complex condition with many unresolved issues. Until today, no guideline or consent exists on how exactly to diagnose this condition, nor how best to treat it. Currently, the histological finding of infiltration of multiple plasmacytes into the endometrial stroma is considered the gold standard for CE diagnosis (9), but the amount of cells per sample/area or field remains unsettled (10). Based on the different diagnostic methods and investigated population, the prevalence of CE in infertile women varies considerably among different studies, from 2.8 to 86.5% (11–13). Interestingly, the incidence rate of CE was reported even higher, namely, ranging from 14 to 67.5% for women with RIF (5–7, 14–16) and 9.3–67.6% for recurrent miscarriage (RM) (3, 8, 12, 17, 18). Despite antibiotics being the primary prescription for CE, depending on the infectious agent detected and on the antibiogram result, the types, dosages, durations, and routes were inconsistent (19). Therefore, the cure rates of CE were reported to range from 52.94 to 100% after antibiotic therapy in previous studies (6, 8, 11, 12, 20). Some publications suggest that the administration of oral antibiotics could improve reproductive outcomes (6–8). The question of whether antibiotics are appropriate in the cure and relevant for pregnancy outcomes in patients with CE is important and still not completely clarified. For this reason, the aims of our systematic review and meta-analysis are to evaluate the reproductive effects of antibiotic treatment for chronic endometritis (CE) in women with RIF or RPL.

## Materials and methods

### Search strategy

Literature searches were performed using three electronic databases (PubMed, Embase, and Web of Science) until 1 December 2021 (without date restriction). Key search terms were as follows: (chronic endometritis OR endometrial inflammation OR CD138 OR plasma cells) AND (infertility OR repeated implantation failure OR repetitive implantation failure OR recurrent implantation failure OR recurrent pregnancy loss OR recurrent miscarriage OR recurrent spontaneous abortion). We also did a manual search to avoid missing relevant publications from the reference lists of key articles.

### Eligibility criteria

The inclusion criteria were as follows: (1) experimental or observational studies in the English language; (2) participants who experienced reproductive failures, including infertility, recurrent implantation failure (RIF), and recurrent pregnancy loss (RPL); (3) all women who underwent diagnostic hysteroscopy and endometrial biopsy for histological analysis to confirm CE; and (4) all women who received assisted reproductive technology (ART) or attempted spontaneous pregnancy were monitored the reproductive outcomes.

The exclusion criteria were as follows: (1) studies without complete data; (2) studies such as case reports and reviews; and (3) studies evaluating other types of endometrial inflammation (e.g., acute, subacute, or tubercular endometritis).

### Study selection and data extraction

Two investigators independently reviewed the inclusion criteria to select articles that qualified. Any disagreement was resolved through discussions with a third reviewer. Two investigators independently extracted the outcome data and study characteristics from eligible studies using piloted screening forms in Microsoft Office Excel. The results were examined repeatedly and discrepancies were discussed until a consensus was reached.

#### Comparators

Comparators were as follows: (1) Women with treated CE vs. untreated CE: defined as women receiving antibiotic treatment for CE vs. women with CE not receiving antibiotics. Control biopsy was not performed. (2) Women with cured CE vs. non-CE: defined as women with CE resolution (after antibiotic therapy) vs. women negative for CE (with normal endometrial histology). (3) Women with cured CE vs. persistent CE: defined as women in whom (after antibiotic therapy) a control biopsy showed the resolution of CE vs. those in which CE was still present.

#### Outcomes

Outcomes were on-going pregnancy or live birth rate [per patient (OPR/LBR)]: “on-going pregnancy” was defined as maintenance of pregnancy at 12 weeks or later of gestation; “live birth” was defined as a birth of at least one newborn after 24 weeks of gestation; clinical pregnancy rate [per patient (CPR)] was defined as the appearance of an intrauterine gestational sac with positive cardiac movement as documented by *trans*-vaginal ultrasonography (21); miscarriage rate or pregnancy loss rate [per clinical pregnancy (MR/PLR)] was defined as a pregnancy loss before 24 weeks of gestation.

### Risk of bias

The quality assessment of all included studies was implemented by two reviewers based on the Newcastle-Ottawa Scale (NOS) for observational studies.

### Statistical analysis

The meta-analysis was performed using Review Manager version 5.4.1 (Nordic Cochrane Centre, Cochrane Collaboration). All outcomes were compared, and any differences were discussed. Study outcomes were expressed using an odds ratio (OR) with a 95% confidence interval (95% CI). A *P*-value of <0.05 was defined as indicative of a statistically significant difference in results. Heterogeneity was assessed by presenting forest plots and by calculating the *I*^2^ value (>50% was considered extensive heterogeneity). If only *I*^2^ < 50%, heterogeneity was acceptable. When heterogeneity was high, a random-effects model was used to estimate study results; otherwise, the fixed-effects meta-analysis was performed. Potential publication bias was also illustrated qualitatively with a funnel plot using the Rev Man software if the distribution of CIs was significantly different.

## Results

### Study inclusion and basic characteristics

**FIGURE 1:**
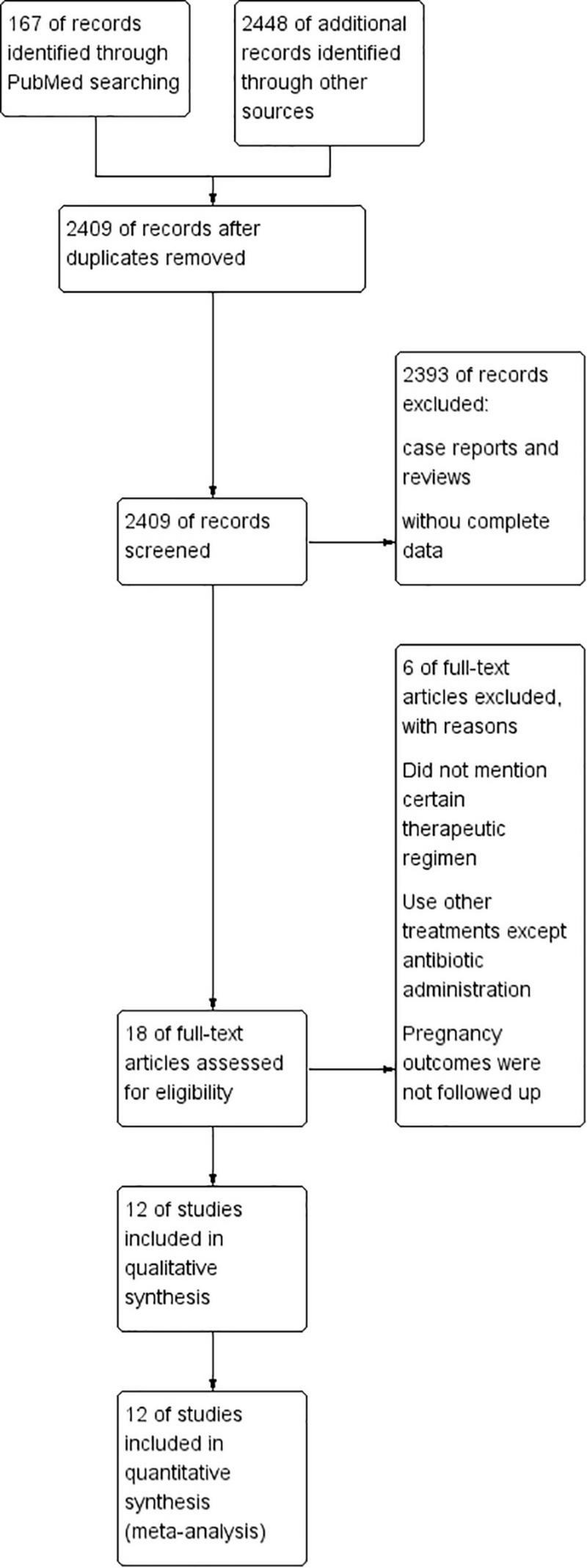
Study flow diagram.

The search strategy initially retrieved 2,615 potentially relevant publications (PubMed: 167, EMBASE: 237, and Web of Science: 2,211). After removing duplicates, the titles and abstracts of the remaining 2,409 records were screened. Then, 18 studies were preselected for inclusion. After an assessment of the eligibility criteria, six articles were excluded (they did not mention certain therapeutic regimens; used other treatments except antibiotic administration; pregnancy outcomes were not well followed up). Finally, a total of 12 studies (3, 6–8, 12, 14–18, 20, 22) were included in the present meta-analysis (Figure 1).

With respect to the study design, most studies included in this review were observational studies, of which six were retrospective studies, five were prospective studies, and one was a case-control study. The detailed baseline characteristics of the included studies are presented in Table 1.

**TABLE 1:** General characteristics of the included studies.

	Study design	Subjects	Group	Diagnosis criterion	Antibiotic treatment
Demirdag et al. (14)	Retrospective study	Recurrent implantation failure (RIF) patients	Group A: Patients diagnosed with CE and treated by antibiotics (*n* = 129); Group B: Patients without CE (*n* = 103)	CD138 + ≥ 1 ↑/HPF	Oral Ciprofloxacin 500 mg twice per day and oral Ornidazole 500 mg twice per day for 14 days.
Song et al. (20)	Prospective, single-blind randomized controlled trial	Women with reproductive failure including infertility, recurrent miscarriage, or RIF.	Group A: Antibiotic treatment group (*n* = 59); Group B: No- treatment group (*n* = 55)	Positive when ≥ 1 plasma cell was identified per 10 HPF	Oral levofloxacin 500 mg and tinidazole 1,000 mg daily for 14 days
Gay et al. (3)	Monocentric retrospective comparative study	42 patients consulting for repeated pregnancy losses (including early or late miscarriages)	Group A: No endometritis (*n* = 20); Group B: Treated endometritis (*n* = 13); Group C: Untreated endometritis (*n* = 9).	The existence of at least one plasma cell per field on endometrial biopsy.	Germ-oriented antibiotic therapy or with doxycycline (100 mg × 2/day) and metronidazole (500 mg × 2/day) by mouth for 14 days in other cases
Kitaya et al. (6)	Observational cohort study using prospectively collected data	438 infertile women with a history of RIF	Group A: Women with cured CE (*n* = 116), Group B: Women with persistent CE (*n* = 4); Group C: Women without CE (*n* = 226).	The endometrial stromal plasmacyte density index (ESPDI) = CD138+/ 20 HPF, CE was diagnosed as 0.25 or more ESPDI.	T1. Doxycycline (200 mg/day for 14 days) T2. Ciprofloxacin and metronidazole (500 mg of each for 14 days)
Cicinelli et al. (12)	Retrospective study	A total number of 95 women with unexplained infertility	Group A: Patients negative for CE (*n* = 42); Group B: Patients with initial diagnosis of CE and subsequent CE resolution after treatment (*n* = 38); Group C: Patients with persistent CE after treatment (*n* = 15).	Hysteroscopy; the presence of 1–5 plasma cells/HPF or discrete clusters of <20 plasma cells by CD138 staining	An appropriate antibiotic treatment
McQueen et al. (18)	Case-control observational study	A total of 107 women with two or more pregnancy losses	Group A: Women with treated for CE (*n* = 17); Group B: Women with untreated CE (*n* = 51).	The presence of 1–5 plasma cells/HPF or discrete clusters of <20 plasma cells by CD138 staining	Empiric treatment with doxycycline (100 mg two times per day) for 14–21 days
Cicinelli et al. (12)	Retrospective cohort study	106 women with unexplained infertility and a history of RIF.	Group A: Women with cured CE (*n* = 46), Group B: Women with persistent CE (*n* = 15);	Hysteroscopy; histology; the presence of microorganisms	T1. Doxycycline (200 mg/day for 14 days) T2. Ciprofloxacin and metronidazole (500 mg of each for 14 days)
Tersoglio et al. (22)	Prospective study of a model-based control with analogue abductive methodology	30 patients undergoing IVF-ET who had recurrent implantation failure (RIF)	Group A: Women with cured CE (*n* = 9), Group B: Women with persistent CE (*n* = 5); Group C: Women without CE (*n* = 16).	At least ≥ 1 plasma cell/HPF	Doxycycline 200 mg/day for 14 days, continuing in association with metronidazole 1 g/day and ciprofloxacin 1 g/day for 14 days If no remission of the inflammatory process is achieved, the above scheme is repeated, in association with linezolid 600 mg/day orally for 10 days + All the patients received corticosteroid therapy in doses meprednisone orally 4 to 8 daily mg; Glycine 100 mg/day associated with Vit. E 300 mg, Vit. B6 100 mg and Vit. A 10.000 UI/day orally
Cicinelli et al. (17)	Retrospective study	360 women with unexplained RM	Group A: Women with cured CE (*n* = 118), Group B: Women with persistent CE (*n* = 78);	Hysteroscopy (the demonstration of micropolyps that fluctuate in the cavity, stromal edema, and focal or diffuse hyperemia); histology (the presence of 1–5 plasma cells/HPF); the presence of microorganisms	An appropriate antibiotic treatment
McQueen et al. (8)	Observational cohort study using prospectively collected data	Three hundred ninety-five women with a history of two or more pregnancy losses of less than 10 weeks’ size or a fetal demise of 10 or more weeks’ size.	Group A: Women with cured CE (*n* = 24), Group B: Women without CE (*n* = 244);	The presence of plasma cells on endometrial biopsy.	1. Ofloxacin (800 mg) and metronidazole (100 mg) for 2 weeks T2. Doxycycline alone, doxycycline and metronidazole, or ciprofloxacin and metronidazole
Yang et al. (15)	Prospective study	202 consecutive RIF cases with CE histological diagnosis	Group A: Women with treated CE (*n* = 68); Group B: Women with untreated CE (*n* = 20).	Hysteroscopy; HE staining as well as CD38 and CD138 immunohistochemical staining (data extraction source)	2 weeks of levofloxacin 0.5 g qd and metronidazole 1 g qd
Johnston- MacAnanny et al. (16)	Retrospective chart review	Thirty- three patients with recurrent implantation failure (RIF)	Group A: Women with cured CE (*n* = 10) Group B: Women without CE (*n* = 23)	CD138 + ≥ 1 ↑/HPF	T1. Doxycycline (200 mg/day for 14 days) T2. Ciprofloxacin and metronidazole (500 mg of each for 14 days)

#### Population

All studies enrolled 2,154 women with PRL/RM and RIF. Recurrent pregnancy loss (RPL) was defined as the loss of two or more clinically recognized pregnancies occurring before 20–24 weeks of gestation and includes embryonic and fetal losses (23). Recurrent implantation failure (RIF) was defined as failure to achieve a clinical pregnancy after the transfer of at least four good-quality embryos in a minimum of three fresh or frozen cycles in a woman under the age of 40 years (24).

#### Diagnosis of chronic endometritis

Currently, CE is diagnosed by endometrial biopsy, and the presence of plasma cells in the endometrial stroma is the generally accepted histological diagnostic criterion for CE. Plasma cells were identified in the stroma by traditional hematoxylin and eosin (H&E) staining alone. Thus, immunohistochemistry (IHC) for detection of the plasma cell marker CD138 (also known as syndecan-1) is used clinically to diagnose CE since it stains well on the surface of plasma cells. In most studies in this review, the diagnosis was based on the demonstration of at least one CD138-positive plasma cell/HPF. However, in Cicinelli’s studies, the diagnosis of CE was initially based on the demonstration of micropolyps that fluctuate in the cavity, stromal edema, and focal or diffuse hyperhemia, as previously published (7, 12, 17). In the follicular phase of the subsequent cycle following the treatment, all the patients were reevaluated uterine cavity by hysteroscopy for signs of CE and collected endometrial samples for histology and culture (25).

#### Treatment of chronic endometritis

To date, the first-line treatment protocol for CE was oral empiric antibiotics (doxycycline 100 mg two times a day for 14 days; ciprofloxacin and metronidazole 500 mg two times a day for 14 days). However, Cicinelli et al. (12, 17) also selected appropriate antibiotics according to the results of drug sensitivity and administered bacterium-sensitive antibiotics for 2 weeks as the second line. The detailed treatment regimens are presented in Table 1.

### Quality assessment of the risk of study bias

Half of the included studies (*n* = 12) were awarded seven stars, four studies were awarded six stars, and only two studies scored eight stars. The Newcastle-Ottawa Quality Assessment Scale is shown in Table 2.

**TABLE 2:** Results of quality assessment using the Newcastle-Ottawa Scale for the included studies.

Study	Selection				Comparability	Outcomes			Total quality socres
	Representativeness of the exposed cohort	Selection of the non-exposed cohort	Ascertainment of CE	Demonstration that outcome of interest was not present at start of study	Comparability of cohorts on the basis of the design or analysis	Assessment of outcome	Follow-up long enough for outcomes to occur	Adequacy of follow up cohorts	
Demirdag et al. (14)	★	★	_		★★	★	★	★	7
Song et al. (20)	★	★	★		★★	★	★	★	8
Gay et al. (3)	_	★	★		★★	★	★	★	7
Kitaya et al. (6)	★	★	★		★★	★	★	★	8
Cicinelli et al. (12)	★	★	★		★★	_	★	★	7
Cicinelli et al. (7)	★	★	★		★★	★	_	★	7
Tersoglio et al. (22)	★	_	★		★	★	★	★	6
McQueen et al. (18)	★	★	★		★★	★	—	—	6
Cicinelli et al. (17)	★	★	★		★★	_	★	★	7
Yang et al. (15)	_	★	★		★	★	★	★	6
McQueen et al. (8)	★	★	★		★	★	★	★	7
Johnston-MacAnanny et al. (16)	_	★	★		★	★	★	★	6

### Synthesis of results

#### Treated chronic endometritis versus untreated chronic endometritis

**FIGURE 2:**
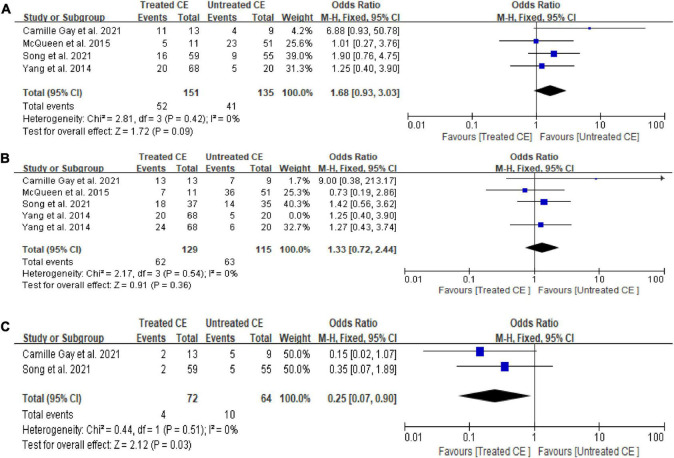
Forest plot of comparison: Treated chronic endometritis vs. Untreated: **(A)** on-going pregnancy/live birth rate; **(B)** clinical pregnancy rate; **(C)** miscarriage rate/pregnancy loss rate. M-H Mantal Haenszel.

Compared with the control group, women with CE receiving antibiotics did not show a statistically significant difference in OPR/LBR (OR = 1.68, 95% CI = 0.93–3.03, *I*^2^ = 0%, *P* = 0.09) and CPR (OR = 1.33, 95% CI = 0.72–2.44, *I*^2^ = 0%, *P* = 0.36), although there was a lower MR (OR = 0.25, 95% CI = 0.07–0.90, *I*^2^ = 0%, *P* = 0.03; Figure 2). Sensitivity analysis was not performed due to minimal inconsistency (*I*^2^ = 0%).

#### Cured chronic endometritis versus non-chronic endometritis

**FIGURE 3:**
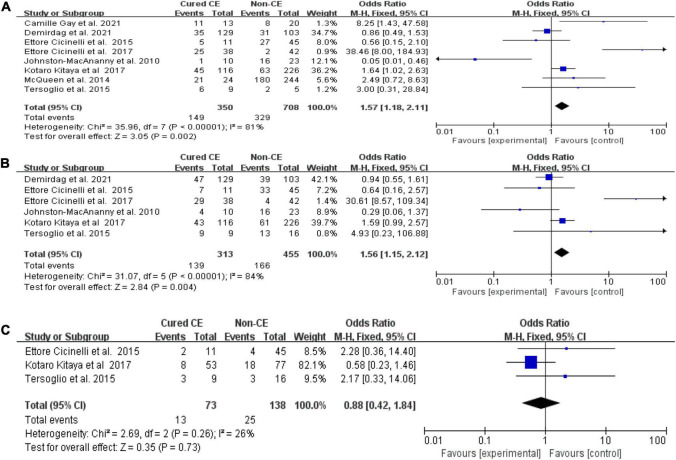
Forest plot of comparison: Cured chronic endometritis vs. Non-chronic endometritis. **(A)** on-going pregnancy/live birth rate; **(B)** clinical pregnancy rate; **(C)** miscarriage rate/pregnancy loss rate. M-H Mantal Haenszel.

We found higher OPR/LBR (OR = 1.57, 95% CI = 1.18–2.11, *I*^2^ = 81%, *P* = 0.002) and CPR (OR = 1.56, 95% CI = 1.15–2.12, *I*^2^ = 84%, *P* = 0.004) in women with cured CE in comparison with those with non-CE, with no difference in terms of MR/PLR (*P* = 0.73; Figure 3). The exclusion of the study by Cicinelli et al. (12) from the pooled analysis did yield significant changes to OPR/LBR (*I*^2^ = 68%, *P* = 0.17) and CPR (*I*^2^ = 42%, *P* = 0.40).

#### Cured chronic endometritis versus persistent chronic endometritis

**FIGURE 4:**
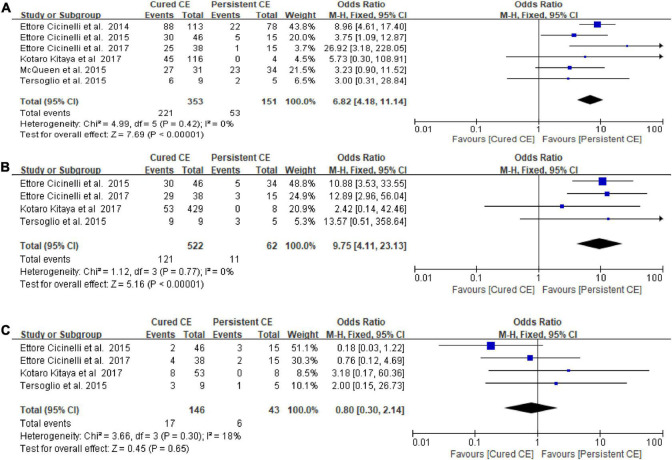
Forest plot of comparison: Cured chronic endometritis vs. Persistent chronic endometritis. **(A)** on-going pregnancy/live birth rate; **(B)** clinical pregnancy rate; **(C)** miscarriage rate/pregnancy loss rate. M-H Mantal Haenszel.

There was a statistically significantly higher OPR/LBR (OR = 6.82, 95% CI = 4.18–11.14, *I*^2^ = 0% *P* < 0.00001) and CPR (OR = 9.75, 95% CI = 4.11–23.13, *I*^2^ = 0%, *P* < 0.00001) in women with cured CE vs. those with persistent CE. No significant differences were found in MR/PLR (OR = 0.80, 95% CI = 0.30–2.14, *I*^2^ = 18%, *P* = 0.65; Figure 4).

## Discussion

### Main findings

In this systematic review and meta-analysis, our results show that there was no statistically significant difference in OPR/LBR and CPR for women with CE receiving treatment vs. those not receiving therapy. Nevertheless, women with cured CE by effective treatment considerably improve in the clinical pregnancy rate and live birth rate/on-going pregnancy rate in comparison with those with persistent CE. Hence, we have considered that a repeat control biopsy should be performed to assess patients for CE resolution. The above findings suggest that CE is associated with adverse reproductive outcomes, such as RIF and RPL, whose accurate evaluation and effective treatment can promote the chance of successful pregnancy and live birth.

### Interpretation and implications

A variety of studies in a population with a poor prognosis (repeated implantation failure and recurrent miscarriage) have suggested that a regimen of oral antibiotics for CE, which is a promising therapeutic strategy, could eliminate endometrium stromal plasma cells (ESPC) and improve reproductive outcomes to some extent (7, 8, 15, 17). However, two studies conclude that reproductive outcomes may not be improved after a single course of oral broad-spectrum antibiotics (14, 20). For one reason, in a study by Song et al. (20), the reproductive outcomes as subordinate endpoints were not found to have an adequately significant effect in distinct discrepancies between the groups (+17.5% OPR and 8.9% MR in the treatment arm vs. controls). Therefore, a further RCT with much larger sample size and a more homogeneous population is needed to be conducted based on a clinically oriented primary endpoint. In contrast, the diagnosis of CE depends on the immunohistochemical detection of plasma cells in endometrial biopsy samples, which produces a methodological bias in the assessment of CE cure. An assessment that was calculated as all CD138+ cell counts in an entire section evaluated divided by the account of the unit area could overcome the problem of local fluctuations in plasmacyte count as well as rectify the variation in results caused by sample size differences (10). Moreover, endometrial biopsy is actually a local scratch or injury to the endometrium, which has been found to improve IVF outcomes and subsequent clinical pregnancy and birth (26–28). In addition, there is a lack of consensus regarding optimal antibiotics, dose, and duration for the treatment of chronic endometritis. In clinical practice, even the microorganism causing the infection is frequently not identified, broad-spectrum antibiotics are usually prescribed, which can contribute to a high rate of recurrent infections after treatment, as well as side effects derived from the clearance of endogenous off-target microbiota in the uterine cavity and other body sites (29). If identification of microorganisms were carried out, antibiotic guidelines could be adapted to the pathogen found and to any possible allergy the patient might have to the antibiotics used (7, 12).

Interestingly, we found that the abortion rate decreased after antibiotic treatment, which may be related to the modification of the endometrial microenvironment. Recurrent pregnancy loss has been related to subclinical infection, endometrial inflammation status, and the abnormal endometrial microenvironment. The presence of CE can modify the receptivity of the endometrium with an abnormal microbiome environment that disturbs normal implantation (30). For successful implantation, mediators of inflammation such as leukocytes, cytokines, chemokines, and other endometrial factors (31–33), which play a crucial role in the regulation of immune status (34) and growth of the trophoblast, may modify endometrial receptivity. CE also alters uterine contractility in both the periovulatory and mid-luteal phases, which could help explain some symptoms such as pelvic pain, AUB, and implantation failure (35). Furthermore, the presence of CE may affect implantation and the establishment of pregnancy through disturbing decidualization *in vitro* and weakening the action of progesterone on endometrial stromal cells (ESC) (36). These findings may offer suggestions for the presence of chronic endometritis before pregnancy, which may be beneficial for future fertility treatment. Consequently, appropriate administration of antibiotics could not only decrease infectious agents for cured histopathologic CE but also be essential to improve endometrial receptivity.

### Strength and limitations

The strength of this study comprises the rigorous design and comprehensive review, with a literature search completed by an information specialist. The characteristics of the included studies were summarized in detail. A particular novelty of our review is that we estimate the effects of therapy for CE in a population with RIF and RPL. In RIF and RPL cases, the accurate detection and therapy of chronic endometritis would avoid the excessive use of unnecessary assisted reproductive tests and could reduce financial uncertainty and shorten the time. Our study may open new cues in promoting future well-designed studies, providing essential information to scientists regarding the design of optimal management of CE diagnosis (and treatment).

There are also several limitations to be considered in this review. Initially, as no agreed gold standard or guidelines for the diagnosis and treatment of CE exists, it is very hard to group the trials according to similar procedures and standards. This also explains the large variety of prevalence presented in different studies. The inconsistent use of endometrial culture and antibiotic regimens (type of drug and duration) as well as different ovarian stimulation protocols and IVF-ET process may cause confounding bias in the results in evaluating the impacts of CE treatment on reproductive outcomes. Additionally, the ascertainment method of chronic endometritis resolution and the times of repeated hysteroscopy and biopsy for histopathologic CD138 immunohistochemical examination until the features were negatively varied among studies, potentially producing a deviation in CE detection. Furthermore, enrolled women with heterogeneous characteristics (Table 1) (i.e., suffering from repeated implantation failure and recurrent miscarriage would potentially lead to diverse estimates of the reproductive outcomes, but it can ensure the generality of results). Finally, what is effectively lacking are randomized clinical trials to improve the quality of analysis.

### Conclusion

The present meta-analysis demonstrates that while antibiotic treatment is a sensible option to cure CE, more well-designed prospective studies are needed to evaluate the reproductive impact of antibiotic treatment. The control biopsy should be performed to confirm CE resolution (at histology). Cured CE provides high-quality maternal conditions for subsequent embryo transfer and successful pregnancy.

## Data availability statement

The original contributions presented in this study are included in the article/supplementary material, further inquiries can be directed to the corresponding authors.

## Author contributions

JL and LY independently reviewed the inclusion criteria to select articles qualified. LC resolved any disagreement through discussion. ZL and YL independently extracted the outcome data and study characteristics from eligible studies using piloted screening forms in Microsoft Office Excel. All authors contributed to the article and approved the submitted version.
